# Correction to “Large B‐cell lymphoma (LBCL): EHA Clinical Practice Guidelines for diagnosis, treatment, and follow‐up”

**DOI:** 10.1002/hem3.70273

**Published:** 2025-12-28

Thieblemont C, Gomes Da Silva M, Leppä S, et al. Large B‐cell lymphoma (LBCL): EHA Clinical Practice Guidelines for diagnosis, treatment, and follow‐up. *HemaSphere*. 2025;9(9):e70207. doi:10.1002/hem3.70207.

The abbreviation CSF was incorrectly defined as “colony‐stimulating factor” in the Abstract and Table 2. The correct definition is “cerebrospinal fluid.”

Additionally, in the author listing of the manuscript, the name of an author was incorrectly listed as Marie‐José Kersten. The correct name is Marie José Kersten.

The original article has been updated. We apologize for these errors.

